# Developing a new Governance Approval Process to support federated discovery and meta-analysis of data across the UK through the CO-CONNECT project.

**DOI:** 10.23889/ijpds.v7i3.1799

**Published:** 2022-08-25

**Authors:** Emily Jefferson, Jenny Johnston, Gordon Milligan, Phil Quinlan

**Affiliations:** 1 University of York; 2 University of Nottingham

## Objectives

To develop a new approval process for federated data custodians to install and support a new platform which enables researchers to run from one website, instantaneous, aggregate-level queries to determine the number of patients in each dataset which meet their research criteria.

To agree security controls across data custodians which protect patient confidentiality whilst also providing this new automated capability for researchers and reducing the burden on each data custodian to manually provide the information.

## Approach

The COVID - Curated and Open aNalysis aNd rEsearCh plaTform (CO-CONNECT) has integrated a Cohort Discovery Tool into the Health Data Research (HDR) UK Innovation Gateway website and is connecting >50 different federated datasets. The underpinning architecture is novel, without precedent at such a scale in the UK. We found that although each data custodian recognised the benefits of the platform, many were unclear of the process to formally approve this new model. We have worked across data custodians to co-develop the required new processes and document the security controls.

## Results

We found vast differences in technical knowledge and infrastructures across different data custodians, especially across small research groups hosting data on consented research cohorts verses larger organisations who host and manage routinely collected data. A model for approvals evolved for these 2 separate groups:

Consented research cohorts: a 2-stage process of a pre-assessment for the need for a DPIA and/or completed DPIA. All returned a positive outcome which deemed no personal identifiable information was being used.

Unconsented population level data: 4 different documents were required each being approved by different committees within each data custodian: DPIA, Data Access Application, Security Risk Assessment, Disclosure Control Assessment.

As the model was novel to many data custodians, we developed many different explainer videos and detailed step by step instructions.

## Conclusion

We recommend a new approvals process for new technologies/models is developed to support initiatives which are not covered by the traditional data access request process. Increased investment in teams which approve data governance and IT security applications which have been overwhelmed by the increased demand for their services to review COVID-19 related projects would be welcomed.

